# Ibrutinib Plus Venetoclax in Relapsed/Refractory Chronic Lymphocytic Leukemia: The CLARITY Study

**DOI:** 10.1200/JCO.19.00894

**Published:** 2019-07-11

**Authors:** Peter Hillmen, Andy C. Rawstron, Kristian Brock, Samuel Muñoz-Vicente, Francesca J. Yates, Rebecca Bishop, Rebecca Boucher, Donald MacDonald, Christopher Fegan, Alison McCaig, Anna Schuh, Andrew Pettitt, John G. Gribben, Piers E.M. Patten, Stephen Devereux, Adrian Bloor, Christopher P. Fox, Francesco Forconi, Talha Munir

**Affiliations:** ^1^ Leeds Institute of Medical Research at St James’s, University of Leeds, Leeds, United Kingdom; ^2^ St James’s Institute of Oncology, Leeds, United Kingdom; ^3^ Cancer Research UK Clinical Trials Unit, Birmingham, United Kingdom; ^4^ Imperial College Healthcare NHS Trust, London, United Kingdom; ^5^ University Hospital of Wales, Cardiff, United Kingdom; ^6^ Vale University Health Board, Cardiff, United Kingdom; ^7^ Beatson West of Scotland Cancer Centre, Glasgow, United Kingdom; ^8^ Oxford University Hospitals NHS Foundation Trust, Oxford, United Kingdom; ^9^ University of Liverpool, Liverpool, United Kingdom; ^10^ Barts Health NHS Trust, London, United Kingdom; ^11^ Kings College Hospital NHS Foundation Trust, London, United Kingdom; ^12^ Christie Hospital NHS Trust, Manchester, United Kingdom; ^13^ Nottingham University Hospitals NHS Trust, Nottingham, United Kingdom; ^14^ University Hospital Southampton NHS Foundation Trust, Southampton, United Kingdom; ^15^ King's College London, School of Cancer & Pharmaceutical Sciences, London, United Kingdom; ^16^ Cancer Sciences Unit, Cancer Research UK and NIHR Experimental Cancer Medicine Centres, University of Southampton, Southampton, United Kingdom

## Abstract

**PURPOSE:**

The treatment of chronic lymphocytic leukemia (CLL) has been revolutionized by targeted therapies that either inhibit proliferation (ibrutinib) or reactivate apoptosis (venetoclax). Both significantly improve survival in CLL and replace chemoimmunotherapy for many patients. However, individually, they rarely lead to eradication of measurable residual disease (MRD) and usually are taken indefinitely or until progression. We present the CLARITY trial that combined ibrutinib with venetoclax to eradicate detectable CLL with the intention of stopping therapy.

**PATIENTS AND METHODS:**

CLARITY is a phase II trial that combined ibrutinib with venetoclax in patients with relapsed or refractory CLL. The primary end point was eradication of MRD after 12 months of combined therapy. Key secondary end points were response by International Workshop on CLL criteria, safety, and progression-free and overall survival.

**RESULTS:**

In 53 patients after 12 months of ibrutinib plus venetoclax, MRD negativity (fewer than one CLL cell in 10,000 leukocytes) was achieved in the blood of 28 (53%) and the marrow of 19 (36%). Forty-seven patients (89%) responded, and 27 (51%) achieved a complete remission. After a median follow-up of 21.1 months, one patient progressed, and all patients were alive. A single case of biochemical tumor lysis syndrome was observed. Other adverse effects were mild and/or manageable and most commonly were neutropenia or GI events.

**CONCLUSION:**

The combination of ibrutinib plus venetoclax was well tolerated in patients with relapsed or refractory CLL. There was a high rate of MRD eradication that led to the cessation of therapy in some patients. The progression-free and overall survival rates are encouraging for relapsed and refractory CLL.

## INTRODUCTION

Chronic lymphocytic leukemia (CLL) is the most common hematologic malignancy in the Western world, with an incidence of six per 100,000 per year.^1^ A proportion of patients have indolent disease and never require therapy.^2^ In contrast, other patients’ disease is more aggressive and results in significant ill health and reduced life expectancy. The median survival for the whole CLL population is approximately 10 years from diagnosis. Chemoimmunotherapy (CIT), such as fludarabine, cyclophosphamide, and rituximab (FCR) and bendamustine and rituximab (BR), result in remission in most patients. However, CIT is rarely curative, and the majority of patients experience relapse and eventually succumb to their disease. CIT is also associated with toxicity, which leads to significant immediate and late complications (including possible death) and limits its use across the whole patient population.^3,4^

In CLL, malignant B cells proliferate excessively through B-cell receptor (BCR)–dependent signaling and fail to undergo apoptosis efficiently as a result of overexpression of the anti-apoptotic protein B-cell lymphoma 2 (Bcl-2). This dual pathophysiology leads to the accumulation of CLL cells and, thereby, progressive immune dysfunction and tissue infiltration. However, the outlook for patients with CLL has improved dramatically with therapies that directly target components of the BCR signaling pathway,^5^ particularly Bruton tyrosine kinase (Btk), or apoptosis through targeting the Bcl-2 protein.

Ibrutinib is an orally bioavailable irreversible inhibitor of Btk that blocks BCR signaling to prevent CLL cell proliferation and inhibit CLL cell migration and adhesion.^6,7^ Ibrutinib monotherapy is effective in CLL^5^ and leads to rapid reduction in lymphadenopathy and disease redistribution into the peripheral blood (PB).^8-10^ Ibrutinib has been approved by the US Food and Drug Administration (FDA) and European Medicines Agency (EMA) as a single agent for patients with previously untreated and relapsed CLL and in combination with BR in previously treated CLL because it leads to a prolongation of both progression-free and overall survival in both groups.^9,11-15^ Eradication of detectable CLL with single-agent ibrutinib is rare, and patients usually remain on ibrutinib indefinitely or until disease progression.^12-15^ In addition, ibrutinib leads to well-documented adverse effects in a proportion of patients, including diarrhea, fatigue, bruising and hemorrhage, hypertension, and atrial fibrillation, with approximately 10% of patients discontinuing the drug as a result.^11-13,15^

Venetoclax is an orally bioavailable small-molecule inhibitor of Bcl-2 that leads to CLL cell apoptosis.^16,17^ Venetoclax therefore would be expected to sensitize CLL cells to death by other discrete mechanisms. In early studies, venetoclax showed unexpected efficacy as monotherapy, with a proportion of patients with high-risk CLL achieving eradication of measurable residual disease (MRD) to either venetoclax monotherapy^18^ or the combination of venetoclax with rituximab.^19^ Venetoclax as a single agent is approved by the FDA for patients with CLL who have received at least one prior therapy and by the EMA for previously untreated patients with CLL in the presence of chromosome del(17p) or TP53 mutations and for patients with relapsed CLL with or without del(17p) or TP53 mutations who experience treatment failure with a BCR pathway inhibitor and CIT. Both the FDA and the EMA have approved venetoclax in combination with rituximab in patients with relapsed CLL who have received at least one prior treatment. Venetoclax is generally well tolerated but can lead to GI adverse effects (nausea and diarrhea) and neutropenia. The most common adverse events (AEs) experienced at grade 3 and higher are neutropenia, infection, and anemia.^20^ Tumor lysis syndrome (TLS) occasionally occurs in the first month of venetoclax, but with initial dosing commencing at 20 mg/d and ramping up each week to the full dose of 400 mg/d, biochemical TLS occurs in 5% of patients, and clinical TLS is rare.^20,21^ However, the observation that a proportion of patients, probably approximately 15% with monotherapy,^22^ achieve eradication of MRD is encouraging and suggests that patients may be able to stop venetoclax after a defined duration of therapy.

Given the dual pathogenesis of CLL, we hypothesized that the combination of ibrutinib with venetoclax would be expected to be additive or possibly synergistic and would lead to a higher proportion of patients achieving an MRD-negative remission and therefore being able to stop therapy. The CLARITY trial is a phase II single-arm study to investigate the safety and efficacy of combining ibrutinib with venetoclax in patients with CLL who have either progressed during or after conventional CIT (FCR or BR) or for patients with chromosome del(17p) who have experienced treatment failure with at least one prior line of therapy.

## PATIENTS AND METHODS

### Study Conduct

CLARITY is a single-arm, phase II study in 54 patients with relapsed/refractory CLL. The study was approved by the National Research Ethics Committee and regulatory review bodies. The review boards of participating institutions approved the study protocol (Data Supplement), which was conducted according to the Declaration of Helsinki and Good Clinical Practice. All patients provided written informed consent. An independent data monitoring committee reviewed safety data throughout the trial. The study was run independently through the National Cancer Research Institute CLL clinical study group and sponsored by the University of Birmingham. Data were collected by investigators and analyses conducted by the study statistician and investigators.

### Patients, Investigations, and Treatment

Patients with CLL who required therapy according to the 2008 International Workshop on Chronic Lymphocytic Leukemia (iwCLL) guidelines,^23^ who had been treated previously with CIT (FCR or BR), or who had chromosome del(17p) and experienced treatment failure with at least one line of therapy were recruited. Prior treatment with idelalisib was allowed. Patients previously treated with either ibrutinib (or an alternative Btk inhibitor) or venetoclax were excluded, as were patients with significant comorbidity, previous Richter transformation, CNS involvement, or active autoimmune complications. Creatinine clearance had to be greater than 50 mL/min. Full eligibility criteria are provided in the Data Supplement.

AEs were assessed at protocol-specified time points from day 1 of treatment until 30 days after the end of therapy according to National Cancer Institute Common Terminology Criteria for Adverse Events (version 4). AEs that met the definition of seriousness could be reported after the 30-day cutoff at investigator discretion. Dose modification guidelines are included in the Data Supplement.

Patients were initially treated with 8 weeks of ibrutinib monotherapy (420 mg/d). Four patients who discontinued ibrutinib as a result of toxicity did not start combination therapy with venetoclax and were replaced. Fifty patients started venetoclax in combination with ibrutinib on day 1 of week 9. In the first three patients, venetoclax was added at a starting dose of 10 mg/d, with a weekly dose ramp-up to 20 mg, 50 mg, 100 mg, 200 mg, and the maximum dose of 400 mg/d. No TLS was seen for these three patients, so according to the protocol, all subsequent patients began venetoclax at 20 mg/d. Before starting venetoclax, patients were assessed for risk of TLS (Data Supplement) and categorized as low, medium, or high risk. High-risk patients were admitted for the first two doses of venetoclax.

PB and bone marrow (BM) MRD assessments, clinical assessments, and computed tomography (CT) scans were performed at screening (before ibrutinib), week 8 (before venetoclax), month 8 (6 months of ibrutinib plus venetoclax), month 14 (12 months of ibrutinib plus venetoclax), and month 26 (24 months of ibrutinib plus venetoclax). PB and BM MRD were assessed by highly sensitive multiparameter flow cytometry using an assay capable of detecting one CLL cell in 100,000 leukocytes.^24^ The methods used are described in the Data Supplement.

### Treatment and End Points

The primary end point of the trial was the eradication of MRD to fewer than one CLL cell in 10,000 leukocytes (MRD4) according to the 2008 iwCLL guidelines^23^ in both PB and BM after 12 months of ibrutinib plus venetoclax (month 14). Secondary end points were the eradication of MRD (below MRD4) in PB and BM after 6 months of ibrutinib plus venetoclax (month 8) and 24 months of ibrutinib plus venetoclax (month 26). MRD was assessed in a single central laboratory (Haematological Malignancy Diagnostic Service, Leeds, United Kingdom). Other secondary end points were investigator-assessed response by iwCLL criteria (including measurement of lymph node response by CT scan), progression-free and overall survival, and toxicity of ibrutinib plus venetoclax.

The duration of therapy was defined by the confirmed MRD response with the following three possibilities: MRD4 in both PB and BM at month 8 to stop ibrutinib and venetoclax at month 14, MRD detectable at month 8 but MRD4 in both PB and BM at month 14 and/or at month 26 to stop ibrutinib and venetoclax at month 26, and MRD detectable at month 26 to stop venetoclax but continue ibrutinib until progression. Confirmation of MRD response was clinically defined by three consecutive PB samples below MRD4, with the last concurrently confirmed by a BM below MRD4. In addition to confirmation of MRD response, patients were assessed by iwCLL 2008 criteria and confirmed to have a complete response (CR) before stopping therapy.

### Statistical Analyses

The primary assumption was that after 12 months of ibrutinib plus venetoclax, at least 30% of patients would have MRD eradication (PB and BM) according to iwCLL criteria^23^ (MRD4). An A’Hern design^25^ was used with a one-sided statistical significance (α) of 2.5% and statistical power of 95.5% to test the null hypothesis that the rate of eradication is no greater than 10% against the alternative that it exceeds 30%. Thus, if at least 10 of 50 patients achieved MRD eradication in both the PB and the BM, then the combined treatment would be considered of interest for additional investigation. Data were frozen on November 5, 2018.

## RESULTS

### Study Population

Fifty-four patients were recruited from May 2016 to November 2017 (Table 1). The median number of prior therapies was one (range, one to six therapies), including FCR or BR in 45 patients (83%) and idelalisib-containing treatments in 11 (20%). Eleven (22%) of 50 patients had del(17p), nine (20%) of 45 patients had del(11q) but not del(17p), and 40 (75%) of 53 patients had unmutated *IGVH* genes. Four patients stopped ibrutinib because of AEs in the first 8 weeks before starting venetoclax. The reasons for discontinuation were *Aspergillus* infection (brain abscess, grade 3), mucosal infection (grade 1), postural hypotension (grade 2), and multiple intolerances (grade 1 arthralgia, diarrhea, and dizziness). The remaining 50 patients completed the dose ramp-up of venetoclax combined with ibrutinib (Data Supplement).

**TABLE 1.**
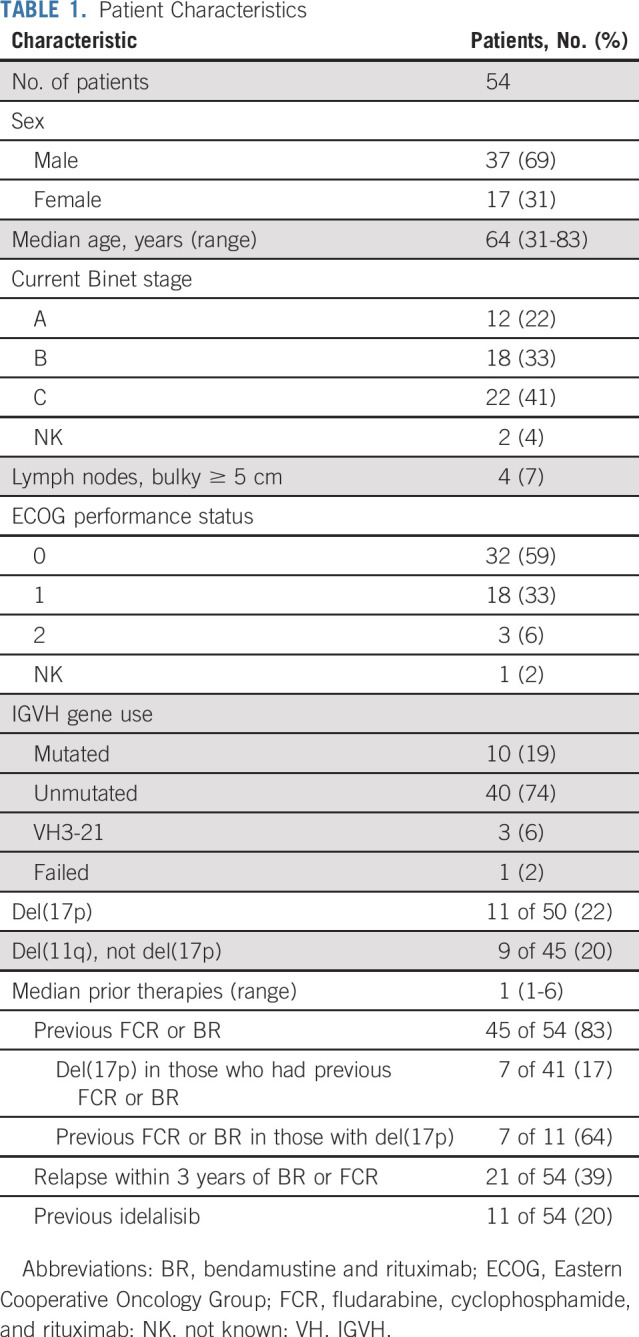
Patient Characteristics

### Safety and Tolerability

A single biochemical TLS event (grade 3) was reported with an increase in both creatinine and phosphate. Dosing of venetoclax was interrupted until the biochemical abnormalities resolved, and the patient subsequently ramped up to 400 mg/d of venetoclax with no additional TLS. At the data lock on November 5, 2018, two suspected unexpected serious adverse reactions, 36 serious AEs, 99 grade 3 or 4 AEs (Tables 2 and 3), and 1,049 AEs (all grades; Data Supplement) were reported. Of note, there were nine grade 3 or 4 infections and 34 episodes of grade 3 or 4 neutropenia. Thus far, all serious AEs have resolved with appropriate management, and all patients remain in the study after resolution. No AEs have been fatal.

**TABLE 2.**
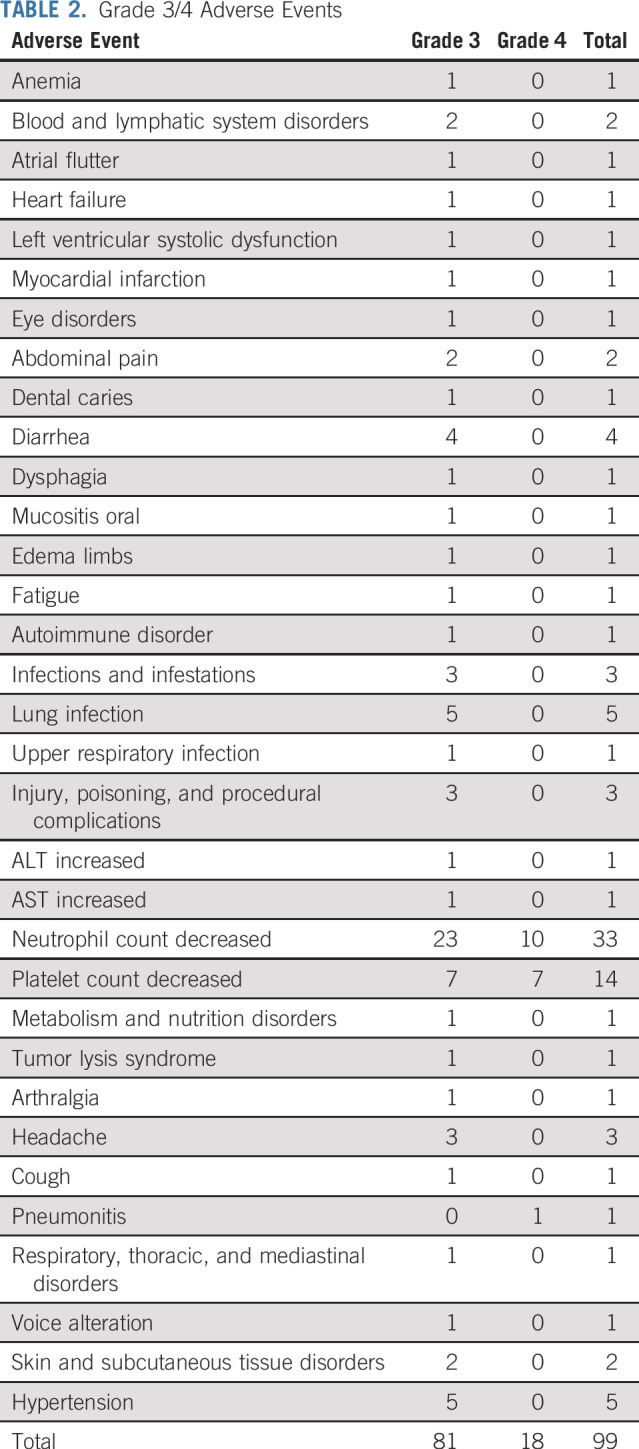
Grade 3/4 Adverse Events

**TABLE 3.**
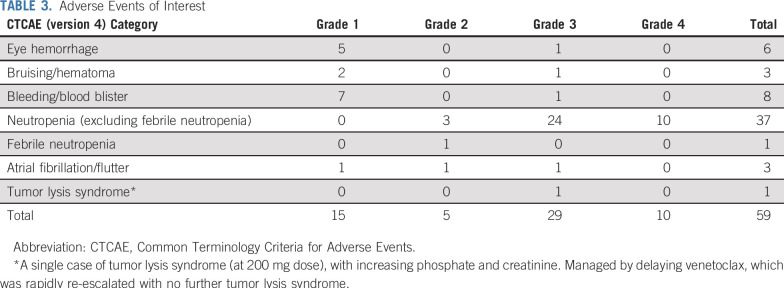
Adverse Events of Interest

Ibrutinib treatment was interrupted in 28 patients for a median of 9 days (range, 1 to 80 days) and reduced in eight patients for a median of 6 days (range, 5 to 121 days). Venetoclax was interrupted in 23 patients for a median of 8 days (range, 1 to 62 days) and reduced in 11 patients for a median of 15 days (range, 3 to 78 days). The majority of treatment modifications for each drug were associated with toxicity; diarrhea and decreased neutrophil count were the most frequently cited AEs.

### Efficacy

**FIG 1.**
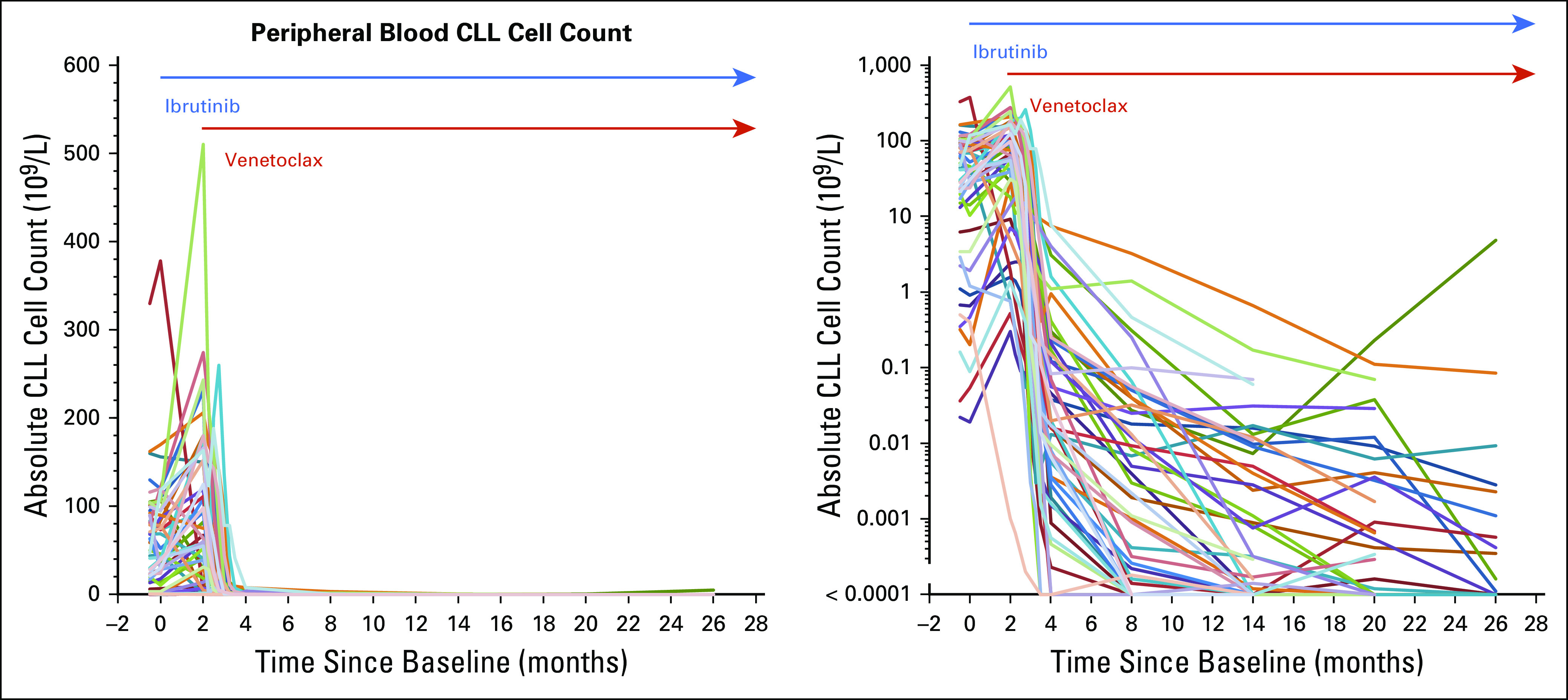
Dynamic of response. CLL, chronic lymphocytic leukemia.

As expected, there was an increase in the level of CLL in PB (Fig 1) during the first 8 weeks of ibrutinib therapy (median absolute increase, 27.3 × 10^9^/L; range, 376 × 10^9^/L decrease to 404 × 10^9^/L increase). Figure 1 shows a rapid response to ibrutinib in all patients, with the level of CLL dropping rapidly again when venetoclax was added and a sustained response in all but one patient.

Forty-nine patients had reached the 14-month (12 months of ibrutinib plus venetoclax) PB, BM, and CT scan assessments at the time of the data freeze. In the analysis of these patients, and the treatment of the four patients who did not commence venetoclax as nonresponders, 47 (89%) of the 53 had an overall response, and 27 (51%) achieved a CR or CR with incomplete BM recovery. Twenty patients (38%) achieved a partial response (11 had lymphadenopathy [generally small residual nodes on a CT scan], two had BM involvement, two had no trephine, three were MRD positive in PB and/or BM, and two were unconfirmed).

**FIG 2.**
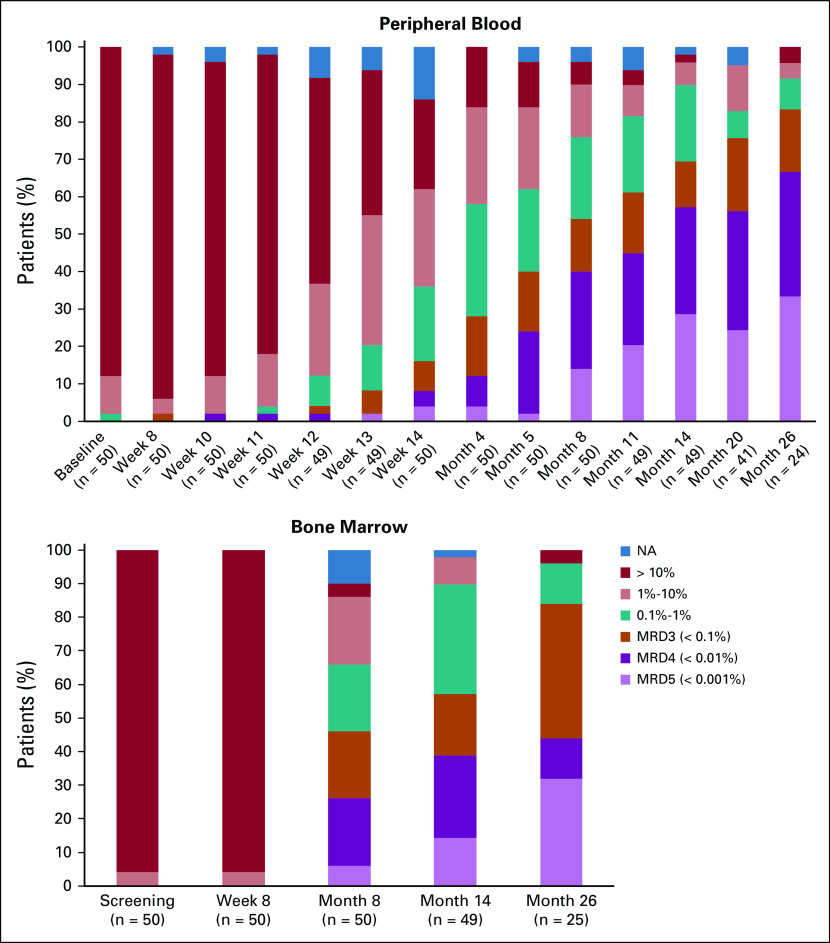
Peripheral blood and bone marrow responses with time. MRD3, fewer than one CLL cell in 1,000 leukocytes; MRD4, measurable residual disease to fewer than one chronic lymphocytic leukemia cell in 10,000 leukocytes; MRD5, residual disease undetectable below a threshold of one CLL cell in 100,000 leukocytes; NA, not available.

The primary end point was the proportion of patients with MRD-negative BM (defined according to iwCLL criteria as MRD4 in the BM at month 14 after 12 months of combined ibrutinib plus venetoclax). This was achieved in 19 (36%) of 53 patients, which thus exceeded the assumption that the combination would be of interest if an excess of 30% of patients became MRD negative. Twenty-eight (53%) of 53 patients were MRD negative in the PB at month 14. Thirty-nine (81%) of 48 patients had no morphologically evident CLL in the BM biopsy (Table 4). Continuous improvement was seen in the depth of MRD reduction, with 11 (44%) of 25 patients achieving MRD4 or below by flow cytometry at month 26 (Fig 2).

**TABLE 4.**
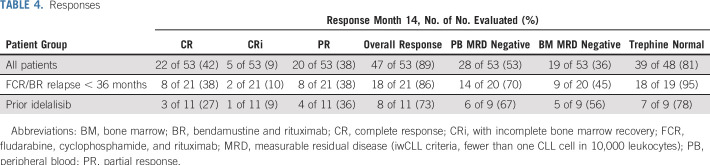
Responses

At the time of the data freeze, two patients were reported to have stopped ibrutinib plus venetoclax after confirmation of MRD-negative remission at month 14. These patients since have not experienced relapse and remain below MRD4 in PB and BM. The remaining patients are continuing therapy within the trial protocol, including those MRD-negative patients with residual lymphadenopathy.

After a median follow-up of 21.1 months, only one patient (adverse IGHV [subset #2/VH3-21], no TP53/ATM deletion, not MRD negative at any point) has had progressive CLL, and no patients developed transformed disease. All 50 patients were alive at the latest follow-up (Data Supplement).

## DISCUSSION

In a study by Ahn et al^26^ with a 5-year follow-up, the overall response rate (ORR) for ibrutinib monotherapy in relapsed CLL was 83% early in the treatment but increased to 95% with prolonged treatment; this includes a minority of patients with persistent lymphocytosis, but no patients achieved an MRD-negative remission after 24 months of ibrutinib. In another study, the ORR for venetoclax monotherapy in relapsed poor-risk CLL was 82%, with 10% of patients achieving a CR.^27^ MRD response for venetoclax monotherapy in del(17p) CLL was reported by Stilgenbauer et al^22^ as 30% MRD negative in the PB at 12 months, but there were limited BM data for confirmation. The combination of ibrutinib plus venetoclax has been investigated in mantle cell lymphoma, with a 71% ORR at week 16 and 75% progression-free survival rate at 12 months, a much better outcome than reported for ibrutinib or venetoclax monotherapies in that indication.^28^

The current results of ibrutinib plus venetoclax after 12 months of combined therapy in a similar patient population are encouraging, with 89% of patients responding to treatment and 51% achieving a CR or CR with incomplete BM recovery. In addition, 36% of patients achieved an MRD-negative remission after 12 months of combination therapy, which is rarely seen with ibrutinib monotherapy and occurs in a small proportion of patients treated with venetoclax. In the MURANO (Venetoclax Plus Rituximab Compared With Bendamustine Plus Rituximab in Relapsed or Refractory CLL) study, 194 patients with relapsed CLL received venetoclax plus rituximab with an ORR of 93.3% and a CR rate of 26.8%; MRD-negative BM was observed in 27.3% at month 9.^29^

Previous reports have demonstrated that achieving MRD negativity in CLL with a variety of therapies, including chemotherapy, immunotherapy, and transplantation, is associated with improved progression-free and overall survival, regardless of the therapy used to achieve a response.^30^ In the CLARITY study, ibrutinib plus venetoclax seems to be effective in achieving MRD eradication in PB and BM by month 14 in patients with CLL who were refractory to CIT and/or had received prior treatment with idelalisib regardless of poor prognostic features, such as chromosome del(17p) or immunoglobulin mutation status. In CLARITY, only one patient progressed, and all patients were alive at the latest follow-up.

The combination of ibrutinib with venetoclax was well tolerated in relapsed and refractory CLL. There were no significant additional AEs with the combination compared with published data on either drug alone. In particular, the incidence of TLS with the combination was one (2%) of 50 patients, which compares favorably with 10 (18%) of 56 patients for venetoclax monotherapy before the dose ramp-up but similar to one (2%) of 60 patients when dose ramp-up was instituted.^27^ When venetoclax was combined with rituximab in a phase III trial, six (3.1%) of 194 patients experienced TLS.^29^ Therefore, with gradual venetoclax dose ramp-up and careful monitoring, TLS is manageable, even when venetoclax is combined with ibrutinib. The other AEs observed with the combination were as expected with either drug alone, including bruising and bleeding (seen with ibrutinib) and neutropenia (seen with venetoclax). There were two suspected unexpected serious adverse reactions: pemphigus (grade 3) and abdominal pain (grade 3). The majority of the other AEs were manageable without delaying or permanently stopping either therapy.

This is the initial description of the combination of two therapies that target the key pathophysiologic pathways seen in relapsed CLL, namely BCR-associated signaling by ibrutinib and apoptosis through venetoclax targeting of Bcl-2. Both ibrutinib and venetoclax are active in CLL with improved survival; however, as monotherapies, both currently are given until disease progression.^26^ We have demonstrated promising efficacy that indicates potent synergy between ibrutinib and venetoclax for inducing MRD-negative responses with manageable adverse effects. The observation that a significant proportion of patients experience MRD-negative remission indicates that this combination can be given for a limited period and then stopped after patients achieve a deep remission. This observation is critical before taking the MRD-guided approach into larger phase III trials. Whether the combination leads to permanent disease eradication in a subset of these patients remains to be seen.

## References

[1] Haematological Malignancy Research Network: Incidence, 2018. https://www.hmrn.org/statistics/incidence

[2] Shanafelt TD Byrd JC Call TG et al Narrative review: Initial management of newly diagnosed, early-stage chronic lymphocytic leukemia Ann Intern Med 145 435 447 2006 16983131 10.7326/0003-4819-145-6-200609190-00007

[3] Eichhorst B Fink A-M Bahlo J et al First-line chemoimmunotherapy with bendamustine and rituximab versus fludarabine, cyclophosphamide, and rituximab in patients with advanced chronic lymphocytic leukaemia (CLL10): An international, open-label, randomised, phase 3, non-inferiority trial Lancet Oncol 17 928 942 2016 27216274 10.1016/S1470-2045(16)30051-1

[4] Goede V Fischer K Busch R et al Obinutuzumab plus chlorambucil in patients with CLL and coexisting conditions N Engl J Med 370 1101 1110 2014 24401022 10.1056/NEJMoa1313984

[5] Byrd JC Furman RR Coutre SE et al Targeting BTK with ibrutinib in relapsed chronic lymphocytic leukemia N Engl J Med 369 32 42 2013 23782158 10.1056/NEJMoa1215637 PMC3772525

[6] de Rooij MFM Kuil A Geest CR et al The clinically active BTK inhibitor PCI-32765 targets B-cell receptor- and chemokine-controlled adhesion and migration in chronic lymphocytic leukemia Blood 119 2590 2594 2012 22279054 10.1182/blood-2011-11-390989

[7] Herman SEM Mustafa RZ Jones J et al Treatment with ibrutinib inhibits BTK- and VLA-4-dependent adhesion of chronic lymphocytic leukemia cells in vivo Clin Cancer Res 21 4642 4651 2015 26089373 10.1158/1078-0432.CCR-15-0781 PMC4609275

[8] Smith DD Goldstein L Cheng M et al Modeling absolute lymphocyte counts after treatment of chronic lymphocytic leukemia with ibrutinib Ann Hematol 94 249 256 2015 25178517 10.1007/s00277-014-2187-9

[9] Woyach JA Smucker K Smith LL et al Prolonged lymphocytosis during ibrutinib therapy is associated with distinct molecular characteristics and does not indicate a suboptimal response to therapy Blood 123 1810 1817 2014 24415539 10.1182/blood-2013-09-527853 PMC3962160

[10] Herman SEM Niemann CU Farooqui M et al Ibrutinib-induced lymphocytosis in patients with chronic lymphocytic leukemia: Correlative analyses from a phase II study Leukemia 28 2188 2196 2014 24699307 10.1038/leu.2014.122 PMC4185271

[11] Byrd JC Brown JR O’Brien S et al Ibrutinib versus ofatumumab in previously treated chronic lymphoid leukemia N Engl J Med 371 213 223 2014 24881631 10.1056/NEJMoa1400376 PMC4134521

[12] Burger JA Tedeschi A Barr PM et al Ibrutinib as initial therapy for patients with chronic lymphocytic leukemia N Engl J Med 373 2425 2437 2015 26639149 10.1056/NEJMoa1509388 PMC4722809

[13] Byrd JC Furman RR Coutre SE et al Three-year follow-up of treatment-naïve and previously treated patients with CLL and SLL receiving single-agent ibrutinib Blood 125 2497 2506 2015 25700432 10.1182/blood-2014-10-606038 PMC4400288

[14] Shanafelt TD Wang V Kay NE et al A randomized phase III study of ibrutinib (PCI-32765)-based therapy vs. standard fludarabine, cyclophosphamide, and rituximab (FCR) chemoimmunotherapy in untreated younger patients with chronic lymphocytic leukemia (CLL): A trial of the ECOG-ACRIN Cancer Research Group (E1912) Blood 132 LBA-4 2018

[15] O’Brien S Furman RR Coutre S et al Single-agent ibrutinib in treatment-naïve and relapsed/refractory chronic lymphocytic leukemia: A 5-year experience Blood 131 1910 1919 2018 29437592 10.1182/blood-2017-10-810044 PMC5921964

[16] Anderson MA Deng J Seymour JF et al The BCL2 selective inhibitor venetoclax induces rapid onset apoptosis of CLL cells in patients via a TP53-independent mechanism Blood 127 3215 3224 2016 27069256 10.1182/blood-2016-01-688796 PMC4920022

[17] Roberts AW Stilgenbauer S Seymour JF et al Venetoclax in patients with previously treated chronic lymphocytic leukemia Clin Cancer Res 23 4527 4533 2017 28100580 10.1158/1078-0432.CCR-16-0955

[18] Wierda W Chyla B Eichhorst B et al Venetoclax in relapsed/refractory chronic lymphocytic leukemia (CLL) with 17p deletion: Outcome and minimal residual disease (MRD) from the full population of the pivotal M13-982 trial Clin Lymphoma Myeloma Leuk 17 S303 2017

[19] Seymour JF Ma S Brander DM et al Venetoclax plus rituximab in relapsed or refractory chronic lymphocytic leukaemia: A phase 1b study Lancet Oncol 18 230 240 2017 28089635 10.1016/S1470-2045(17)30012-8 PMC5316338

[20] Stilgenbauer S Eichhorst B Schetelig J et al Venetoclax in relapsed or refractory chronic lymphocytic leukaemia with 17p deletion: A multicentre, open-label, phase 2 study Lancet Oncol 17 768 778 2016 27178240 10.1016/S1470-2045(16)30019-5

[21] Davids MS, von Keudell G, Portell CA, et al: Revised dose ramp-up to mitigate the risk of tumor lysis syndrome when initiating venetoclax in patients with mantle cell lymphoma. J Clin Oncol 36:3525-3527, 2018 10.1200/JCO.18.00359 30359156

[22] Stilgenbauer S Eichhorst B Schetelig J et al Venetoclax for patients with chronic lymphocytic leukemia with 17p deletion: Results from the full population of a phase II pivotal trial J Clin Oncol 36 1973 1980 2018 29715056 10.1200/JCO.2017.76.6840

[23] Hallek M Cheson BD Catovsky D et al Guidelines for the diagnosis and treatment of chronic lymphocytic leukemia: A report from the International Workshop on Chronic Lymphocytic Leukemia updating the National Cancer Institute-Working Group 1996 guidelines Blood 111 5446 5456 2008 18216293 10.1182/blood-2007-06-093906 PMC2972576

[24] Rawstron AC Fazi C Agathangelidis A et al A complementary role of multiparameter flow cytometry and high-throughput sequencing for minimal residual disease detection in chronic lymphocytic leukemia: An European Research Initiative on CLL study Leukemia 30 929 936 2016 26639181 10.1038/leu.2015.313 PMC4832072

[25] A’Hern RP Sample size tables for exact single-stage phase II designs Stat Med 20 859 866 2001 11252008 10.1002/sim.721

[26] Ahn IE, Farooqui MZH, Tian X, et al: Depth and durability of response to ibrutinib in CLL: 5-year follow-up of a phase 2 study. Blood 131:2357-2366, 2018 10.1182/blood-2017-12-820910 PMC5969380 29483101

[27] Roberts AW Davids MS Pagel JM et al Targeting BCL2 with venetoclax in relapsed chronic lymphocytic leukemia N Engl J Med 374 311 322 2016 26639348 10.1056/NEJMoa1513257 PMC7107002

[28] Tam CS Anderson MA Pott C et al Ibrutinib plus venetoclax for the treatment of mantle-cell lymphoma N Engl J Med 378 1211 1223 2018 29590547 10.1056/NEJMoa1715519

[29] Seymour J, Kipps T, Eichhorst B, et al: Venetoclax-rituximab in relapsed or refractory chronic lymphocytic leukemia. N Engl J Med 378:1107-1120, 2018 10.1056/NEJMoa1713976 29562156

[30] Kwok M Rawstron AC Varghese A et al Minimal residual disease is an independent predictor for 10-year survival in CLL Blood 128 2770 2773 2016 27697770 10.1182/blood-2016-05-714162

